# A proteomics perspective on 2 years of high-intensity training in horses: a pilot study

**DOI:** 10.1038/s41598-024-75266-8

**Published:** 2024-10-10

**Authors:** L. Johansson, S. Ringmark, J. Bergquist, E. Skiöldebrand, A. Widgren, A. Jansson

**Affiliations:** 1 Department of Animal Biosciences, Swedish University of Agricultural Sciences, P. O. Box 7023, Uppsala, 750 07 Sweden; 2 Department of Chemistry-BMC, Analytical Chemistry and Neurochemistry, Uppsala University, P. O. Box 599, Uppsala, 751 24 Sweden

**Keywords:** Physiology, Biomarkers

## Abstract

The human plasma proteome is rather well studied, but not that of other species, including horses. The aims of this study were to (1), explore differences in plasma proteomic profile of young elite harness trotters kept under standardised conditions and subjected to two different training programmes for 2 years and (2) explore changes in proteomic profile over time during the training period. From September at age 1.5 year to March at age 2 years, 16 Standardbred horses were exposed to the same training programme. In March, high-intensity training was introduced and the horses were divided into two training groups (High and Low). Blood samples were collected at rest in December as 1.5-year-olds, July as 2-year-olds, December as 2.5-year-olds and December as 3.5-year-olds. Untargeted proteomics was performed and a hypothesis-generating approach was used in statistical analysis (t-tests). At the age of 2.5 years, the level of serotransferrin was higher in the High group (*P* = 0.01) and at least at one sampling occasion, proteins associated with fat metabolism, oxidant/antioxidant processes, cardiovascular responses, bone formation and inflammation were lower in High group compared to Low (*P* < 0.05). Analyses of changes over time revealed that levels of proteins involved in energy metabolism, red cell metabolism, circulation, oxidant/antioxidant activity, bone formation, inflammation, immune modulation and cellular and vascular damage changed (*P* < 0.05). The results indicate that proteomics analysis of blood plasma could be a viable tool for evaluation of exercise adaptations, performance and for health monitoring, with several potential biomarkers identified in this study.

**Supplementary Information:**

The online version contains supplementary material available at 10.1038/s41598-024-75266-8.

## Introduction

While the human plasma proteome has been rather well studied in recent years^1^, there have been fewer studies on other species, including horses (*Equus caballus*)^2,3^. Proteomics analysis of blood plasma in horses is an expanding field of research with significant promise for advancing equine health and performance. As with any living organism, the proteins present in a horse’s body play a crucial role in maintaining its overall health and function. These proteins include enzymes, hormones, antibodies and many other proteins, all of which are involved in various physiological processes such as immune response, oxygen transport and blood clotting. Therefore, analysing the proteome of horse plasma offers a unique window into the horse’s internal environment, shedding light on its health status, potential diseases and responses to various environmental and therapeutic factors.

One of the primary applications of proteomics in equine medicine is in disease detection and monitoring^4^. Examples of relevance for race horses are the study by Karagianni et al. (2022) who used tracheal wash to study the development of airway inflammation at the onset of training and the study by Peffers et al. 2015, who identified a set of proteins in synovial fluid that may distinguish normal joints from joints with osteoarthritis. However, collection of organ specific tissues and fluids cannot easily be practiced in a systematic preventive monitoring program, but collection of blood plasma can. By methodically examining the patterns of proteins present in plasma, veterinarians and researchers can identify deviations from baseline that may indicate the presence of underlying illnesses or disorders. Early diagnosis of conditions such as osteoarthritis, airway inflammation or infections becomes more achievable with these advanced analytical techniques, allowing for prompt intervention and improved treatment outcomes. For the equestrian and racing industries, proteomics analysis can also offer insights into optimising the performance of elite equine athletes. Today, there are no molecular biomarkers for evaluating fitness level of horses. By studying the protein markers associated with muscle development, endurance and recovery, trainers and owners can gain valuable information for use in crafting individualised training regimens, nutrition plans and management strategies. The ultimate goal is to optimise each horse’s competitive edge. However, only a few proteomic training studies^5,6^ have been performed in horses and more are needed for a better understanding of the field. As far as we know, until now there is no study on the development of the plasma proteome profile in race horses from the onset of training at the age of 1.5 years and until fit for racing at the age of 3.5 years. Proteomics analysis of blood plasma in horses could provide veterinarians, researchers, trainers and owners with a deeper understanding of the biology and health of their horses, helping to ensure well-being, early disease detection and peak performance. Therefore, as technology continues to advance and knowledge increases, equine proteomics holds great promise for improving the welfare of horses and understanding of their incredible athleticism.

The aims of this study were to (1) explore differences in plasma proteomic profile of young elite harness horses (trotters) kept under standardised conditions and subjected to two different training programmes for 2 years and (2) explore changes in the proteomic profile over time during the training period. The hypotheses were that proteome profile differs between horses in different training programmes and also changes substantially over a 2-year period, and that these changes are reflected in proteome profile. We thus performed untargeted proteomics and applied a hypothesis-generating approach.

## Materials and methods

The study was performed at the Swedish National Centre for Trotting Education at Wången, (Alsen, Sweden) and metabolomics data from the same study and sample collection has earlier been published^65^. The protocol was approved by Umeå Local Ethics Committee, Sweden (A90-10, 2010-09-14) and experiments were performed in accordance with relevant guidelines and regulations. The reporting of the experiments is in accordance with ARRIVE guidelines.

### Horses and management

Sixteen male Standardbred 1.5-year-old trotters from four different breeders were entered into the study in September 2010 and released from the study in December 2012. The horses were all castrated in December 2010 or January 2011. They were housed in individual boxes for about 16 h a day from Monday to Thursday/Friday, while for the rest of the time they were group housed in a paddock with shelter. Their diet consisted of haylage with known energy and nutrient content, which was supplemented with pelleted lucerne (Krafft AB, Malmö, Sweden), a vitamin and mineral supplement (Krafft AB, Malmö, Sweden) and salt (NaCl) to meet the requirements of horses of this age and training intensity^7^. This type of diet is expected to cause greater gut microbial stability^8^and low postprandial insulin fluctuations^9^ compared to diets including cereals. The horses had *ad libitum *access to water and haylage in both the paddock and the box. More information on the nutrient composition of the haylage and on energy and nutritional intake in the horses can be found in our previous publications^10,11^. Body condition was scored (9-point scale) every other month and the horses maintained their body condition (mean score 4.8–5.1) throughout the study^11^.

### Training

From September at the age of 1.5 years to March at the age of 2 years, all horses were exposed to the same training programme. This started with breaking in the autumn and progressed to trotting with a jog cart 4 times/week, with speed gradually increasing up to 5.6 m/s and distance to 5–7 km. In March as 2-year-olds, high-intensity training (i.e. heart rate > 180 bpm, Polar CS600X, Polar Electro, Finland) was introduced and the horses were divided into two training groups, a high training group (High) and a low training group (Low). The groups were balanced with respect to breeder and to parameters known to influence performance, such as age in days, percentage of French ancestry, genetic potential (sire and mean pedigree index estimated with the Best Linear Unbiased Prediction (BLUP) method), inbreeding coefficient, height at withers, conformation, abnormal radiographic findings and proportion of type IIA/type IIB muscle fibres^12^. The training programme for the High group was designed by a group of professional trainers and consisted of two high-intensity training sessions per week, involving heat training, interval training or uphill interval training (Table 1), and 1–2 jogging sessions. The training programme for the Low group was the same as that for the High group, but with a 30% reduction in the high-intensity training distance. For example, during heat training, High horses trotted 1600 m but Low horses only 1100 m (Table 1). Similar velocity was aimed for with both training groups. The mean velocity during heat training was 8.7 ± 0.1 m/s as 2-year-olds and 9.5 ± 0.1 m/s as 3-year-olds. The mean velocity during interval training (flat ground) were 8.3 ± 0.2 m/s as 2-year-olds and 9.3 ± 0.2 m/s as 3-year-olds, and for the uphill interval training mean velocity was 7.3 ± 0.9 m/s. Before and after heat and interval training all horses were warmed up and jogged down for 3,000 m and 1,200 m (~6 m/s), respectively. Before uphill intervals horses were jogged in a hilly terrain (~5 m/s) for 5,500–6,000 m and after the last interval horses were walked (~2 m/s) 500 m back to the stable. More information about the training of the horses can be found in our previous publication^12^.

**Table 1:** Composition of weekly high-intensity training (heart rate > 180 bpm) sessions for 16 Standardbred horses divided into two training groups, high and low (previously described in Johansson et al. 2024).

	High		Low	
Training type	2 years old	3 years old	2 years old	3 years old
Heat	1–2 × 1600 m	2–3 × 1600 m	1–2 × 1100 m	2–3 × 1100 m
Interval	6 × 500–700 m	6 × 700 m	4 × 500–700 m	4 × 700 m
Uphill interval		6 × 600 m		4 × 600 m

### Blood sample collection

Blood samples were collected on four occasions: December as 1.5-year-olds, July as 2-year-olds (14 weeks after high-intensity training was introduced), December as 2.5-year-olds and December as 3.5-year-olds. All blood samples were collected in the early morning (05.00–06.00 h) before any work had started in the stable. The Vacutainer technique was used to draw blood from the jugular vein into lithium heparin tubes (10 mL). Immediately after collection, the blood was centrifuged at room temperature (10 min, 2700 rpm, 920xg) and the plasma was frozen at -20 °C for later analyses.

### Proteomics analysis—chemicals and reagents

Acetonitrile (ACN), methanol (MeOH), acetic acid (HAc), formic acid (FA), ammonium bicarbonate (NH_4_HCO_3_) were obtained from Merck (Darmstadt, Germany). Acetone, protease inhibitor cocktail, trifluoroacetic acid (TFA) were purchased from Sigma Aldrich (St. Louis, MO, USA). For tryptic digestion iodoacetamide (IAA), urea and dithiothreitol (DTT) were obtained from Sigma Aldrich and trypsin (Mass spectrometry grade; Promega, Mannheim, Germany). Ultrapure water was prepared by Milli-Q water purification system (Millipore, Bedford, MA, USA).

### Preparation

Total protein concentration in plasma samples was measured using the DC Protein Assay (Bio-Rad Laboratories, California, USA), with bovine serum albumin (BSA) as the standard. Aliquots corresponding to 20 µg of protein were used for digestion. Briefly, 50 µL of digestion buffer (6 M urea, 50 mM NH_4_HCO_3_) was added to the aliquots of samples. 10 µL of 45 mM aqueous DTT was then added to the samples and the mixtures were incubated at 50 °C for 15 min to reduce the disulfide bridges. The samples were cooled to room temperature (RT) and 10 µL of 100 mM aqueous IAA was added before incubating the mixtures for an additional 15 min at room temperature in darkness in order to carabamidomethylate the cysteines. Finally, a volume of 50 µL of 100 mM NH_4_HCO_3_ was added to all samples followed by the trypsin dissolved in 50 mM NH_4_HCO_3_, yielding a final trypsin/protein concentration of 5% (w/w). The tryptic digestion was performed overnight at 37 °C. Prior to MS analysis, the peptides were purified and desalted by Pierce C18 Spin Columns (Thermo Scientific, Massachusetts, USA). These columns were activated by 2 × 200 µL of 50% acetonitrile (ACN) and equilibrated with 2 × 200 µL of 0.5% trifluoroacetic acid (TFA). The tryptic peptides were adsorbed to the media using three repeated cycles of sample loading and the column was washed using 3 × 200 µL of 0.5% TFA. Finally, the peptides were eluted in 3 × 50 µL of 70% ACN and then vacuum centrifuged to dryness using a Speedvac system ISS110 (Thermo Scientific). Prior to nano-liquid chromatography tandem mass spectrometry (LC-MS/MS), the samples were resolved in 40 µL 0.1% formic acid and then further diluted three times.

### LC − MS/MS

The samples were analyzed using a QExactive Plus Orbitrap mass spectrometer (Thermo Fisher Scientific, Bremen, Germany) equipped with a nano-electrospray ion source. The peptides were separated by reversed-phase LC using an EASY-nLC 1000 system (Thermo Fisher Scientific). A set-up of pre-column and analytical column was used. The pre-column was a 2-cm long nano viper (NV)-column (ID 75 mm, 3 mm particle size, C18; Thermo Fischer Scientific), and the analytical column was a 15-cm long NV-Column (ID 75 mm, 2 mm particle size, C18; Thermo Fisher Scientific). The injection volumes were 5 µL and corresponded to approximately 1 µg of tryptic peptides. Peptides were eluted with a 90 min linear gradient from 4 to 100% acetonitrile at 250 nL min^−1^. The mass spectrometer was operated in positive ion mode, acquiring a survey mass spectrum with resolving power 70,000 (full width half maximum), m/z = 400 − 1750, using an automatic gain control target of 3 × 10^6^. The 10 most intense ions were selected for higher-energy collisional dissociation (HCD) fragmentation (25% normalized collision energy) and MS/MS spectra were generated with an AGC target of 5 × 10^5^ at a resolution of 17,500. The mass spectrometer worked in data-dependent mode.

### Mass spectrometry data handling

The acquired data (.RAW-files) were processed in MaxQuant 1.5.1.2 and database searches were performed using the implemented Andromeda search engine. MS/MS spectra were correlated to a FASTA database containing proteins from *Equus caballus* (Horse) extracted from the Uniprot database (release January 2022). A decoy search database, including common contaminants and a reverse database, was used to estimate the identification false discovery rate (FDR). An FDR of 1% was accepted. The search parameters included: maximum 10 ppm and 0.6 Da error tolerances for the survey scan and MS/MS analysis, respectively; enzyme specificity was trypsin; maximum one missed cleavage site allowed; cysteine carbamidomethylation was set as static modification and oxidation (M) was set as variable modification. The search criteria for protein identification were set to at least two matching peptides. The 63 RAW-data files obtained were quantitatively analysed using the quantification software MaxQuant 1.5.1.2. Label free quantification was applied for comparative proteomics. The results for all samples were combined to a total label-free quantification value for each sample.

### Statistical analyses

Proteins which had four or more values above the detection limit per training group (for analyses of training group) or per age/sampling time (for analyses of change over time) were included in the statistical analyses. In the analyses of effects of training group, an F-test was performed for each protein and age/sampling time, to assess whether the samples had equal or unequal variance. If they had equal variance, a two-tailed student’s t-test was performed. If they had unequal variance, a two-tailed Welsh’s t-test was performed. Numbers of proteins included and significant proteins are listed in Table 2. In the analyses of changes over time, a two-tailed paired t-test for each protein was performed. Numbers of proteins included in this test and significant proteins are listed in Table 3. Statistical analyses were performed in Excel (v16.0.5408.1001, Microsoft, 2023). The p-values were adjusted using the false recovery rate (FDR) method^13^, where they were ranked in ascending order and assigned a rank number. Adjusted p-value was calculated as: p-value x (Total number of test/rank number). Differences were considered significant at *p* < 0.05. Both p-values and FDR-adjusted p-values are presented in the results section. Data are presented as fold change, label-free quantification (LFQ) and mean ± SE (Supplementary Material, SM).

**Table 2:** Number of proteins included in statistical analyses of differences between the high and low Standardbred horse training groups at different sampling ages, and number of proteins that differed significantly between the training groups at each age.

Age	1.5 years old	2 years old	2.5 years old	3.5 years old
Proteins, n	120	133	118	128
p-values < 0.05	1	10	1	4
FDR-adjusted p-values < 0.05	0	0	0	0

**Table 3:** Number of proteins included in statistical analyses of changes over time for the different sampling ages of standardbred horses in training, and number of proteins that differed significantly at each age compared with at age 1.5 years.

Age	2 years old	2.5 years old	3.5 years old
Proteins	143	141	141
p-values < 0.05	35	14	43
FDR-adjusted p-values < 0.05	2	2	13

## Results

### Differences between training groups

In mass spectrometry analyses of proteins in blood plasma from the Standardbred horses subjected to the two different training programmes, a total of 252 proteins were identified. Of these, 16 differed significantly in terms of protein levels between the groups after the initial t-test, but after FDR adjustment no protein levels were significantly different (Table 2). When only the t-test was applied, the level of one unknown protein was significantly higher in the High group than in the Low group at 1.5 years of age (Table 4). At 2 years of age, 10 proteins were present in significantly lower levels in High group compared with Low group according to the t-test, while at 2.5 years of age, one protein was present in higher levels in High group compared with Low group. At 3.5 years of age, four proteins were present in significantly lower levels in High group compared with Low group according to the t-test (Table 4). The levels of all proteins analysed in plasma samples from the training groups are shown in SM1.

**Table 4:** Significant changes in plasma protein levels at four ages in 16 Standardbred horses divided into two training groups, high and low. Fold change compares the high group with the low group (value > 1 indicates that the high group had higher levels than the low group, value < 1 indicates that the high group had lower levels).

Protein	Fold change	*p*-value	FDR-adjusted *p*-value
**1.5 years old**			
Unknown protein	1.57	0.04	5.2
**2 years old**			
Afamin	0.86	0.03	0.9
Apolipoprotein E	0.63	0.01	1.6
Apolipoprotein H	0.88	0.04	0.6
Extracellular matrix protein 1	0.81	0.02	1.0
Fibulin-1	0.90	0.05	0.7
Ig^a^ heavy constant mu	0.70	0.03	1.0
Ig^a^ lambda light chain variable region	0.54	0.02	1.0
Protein AMBP^b^	0.86	0.03	0.7
Serpin family D member 1	0.83	0.03	0.6
Serpin family F member 1	0.79	0.05	0.7
**2.5 years old**			
Serotransferrin	1.35	0.01	1.5
**3.5 years old**			
Complement factor H	0.75	0.03	1.2
Complement factor properdin	0.74	0.02	2.0
Haptoglobin	0.72	0.04	1.2
Ig^a^ lambda light chain variable region	0.55	0.02	1.1

^a^Immunoglobulin; ^b^alpha-1-microglobulin/bikunin precursor.

### Analyses of changes over time

**Fig. 1:**
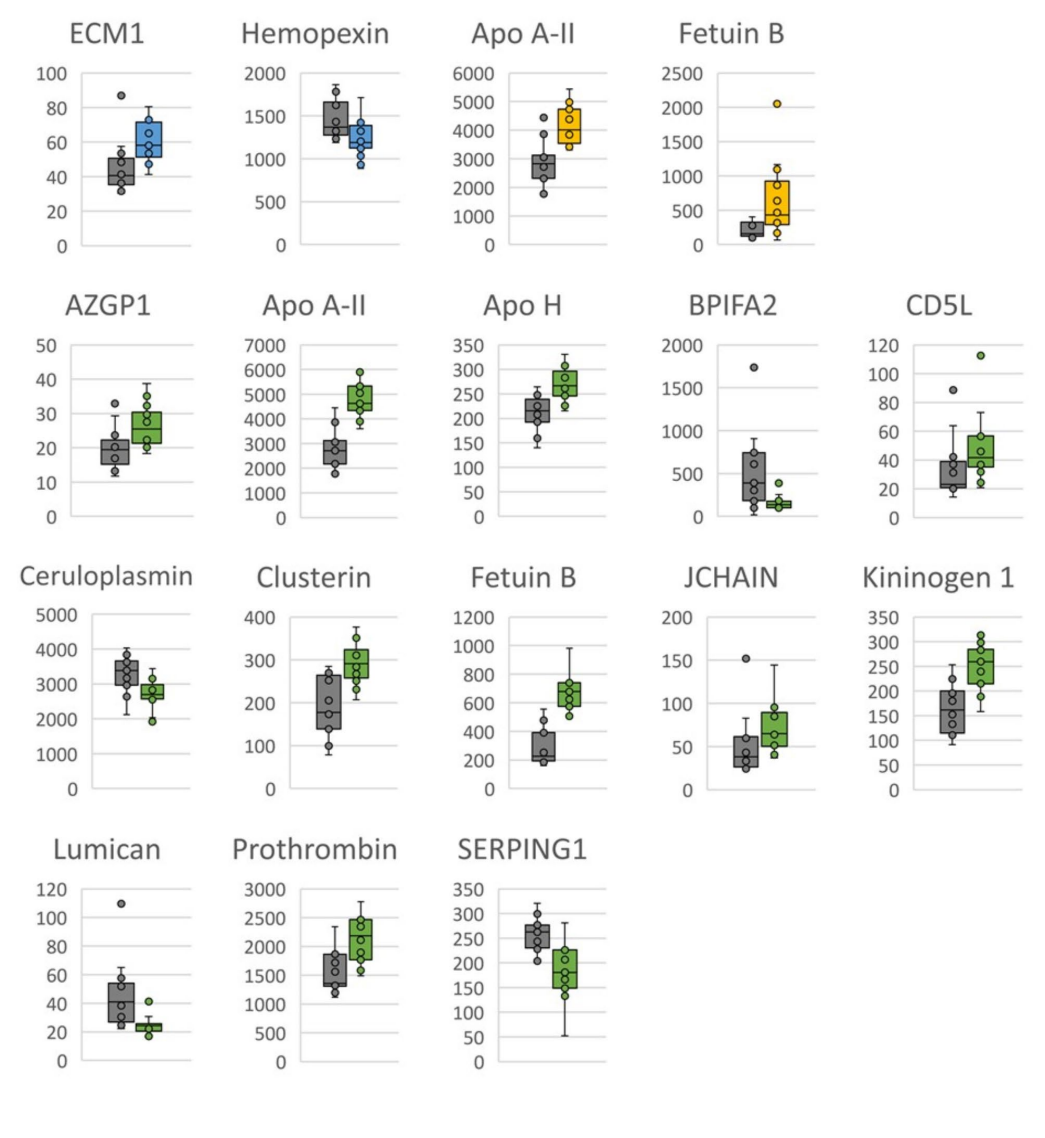
False recovery rate (FDR)-adjusted significantly different plasma protein levels when comparing Standardbred horses at 2 (blue), 2.5 (yellow) and 3.5 (green) years of age with 1.5 years of age (grey). Y-axis shows label-free quantification (LFQ)*10^6^. Abbreviations: alpha-2-glycoprotein 1, zinc-binding (AZGP1), apolipoprotein (Apo), bactericidal/permeability-increasing fold containing family A member 2 (BPIFA2), CD5 molecule like (CD5L), extracellular matrix protein 1 (ECM1), joining chain of multimeric IgA and IgM (JCHAIN), serpin family G member 1 (SERPING1).

Proteomic analyses of changes over time revealed that plasma levels of 90 proteins differed significantly after the initial t-test (Tables 5, 6 and 7), but after FDR adjustment only the levels of 17 proteins remained significantly different (Fig. 1). According to the initial t-test, at 2 years of age 22 proteins were present in significantly higher levels, and 13 proteins in significantly lower levels, compared with 1.5 years of age. After FDR adjustment, the levels of two proteins remained significantly different (Table 5). At 2.5 years of age, 10 proteins were present in significantly higher levels, and four proteins in significantly lower levels, compared with 1.5 years of age, according to the initial t-test, while after FDR adjustment the levels of two proteins remained significantly different (Table 6). At 3.5 years of age, 29 proteins were present in significantly higher levels, and 14 proteins in significantly lower levels, compared with 1.5 years of age, based on the initial t-test. After FDR adjustment, the levels of 13 proteins remained significantly different in that age comparison (Table 7). All changes over time in the different proteins analysed are shown in SM2.

**Table 5:** Significant changes in plasma protein levels at 2 years of age, compared with 1.5 years of age, in 16 Standardbred horses in training. Fold change values > 1 indicate that 2-year-olds had higher levels than 1.5-year-olds, values < 1 indicate that 2-year-olds had lower levels than 1.5-year-olds.

Protein	Fold change	*p*-value	FDR-adjusted *p*-value
2,3-bisphosphoglycerate 3-phosphatase	1.30	0.04	0.2
Afamin	1.14	0.006	0.1
Alpha-1-antitrypsin	0.65	0.01	0.1
Alpha-2-glycoprotein 1, zinc-binding	1.31	0.01	0.1
Amine oxidase	1.21	0.006	0.1
Apolipoprotein C-II	0.32	0.01	0.1
Apolipoprotein E^a^	1.53	0.01	0.1
Apolipoprotein E^b^	1.32	0.02	0.1
Apolipoprotein H	1.20	0.002	0.1
BPI^C^ fold containing family A member 2	0.54	0.03	0.2
C3/C5 convertase	1.09	0.02	0.1
Carboxypeptidase N subunit 2	1.30	0.01	0.1
CD5 molecule like	1.41	0.009	0.1
Complement C1q C chain	0.81	0.04	0.2
Complement C1s	1.18	0.02	0.1
Complement C3	0.57	0.006	0.1
Complement C4-A	0.83	0.03	0.1
Complement component 4 binding protein alpha	1.32	0.01	0.1
Complement component C6	1.19	0.003	0.1
Complement factor H	1.16	0.02	0.1
Extracellular matrix protein 1	1.35	0.0008	0.05
Glutathione peroxidase	0.87	0.05	0.2
Haemopexin	0.85	0.00005	0.007
Histidine-rich glycoprotein	0.72	0.01	0.1
Ig^d^ heavy constant mu	1.23	0.007	0.1
Ig^d^ lambda light chain variable region	0.43	0.05	0.2
Joining chain of multimeric IgA and IgM	1.43	0.008	0.1
Kininogen 1	1.24	0.02	0.1
Lymphocyte cytosolic protein 1	0.52	0.02	0.1
Plasminogen	1.16	0.04	0.2
Protein AMBP^e^	1.40	0.004	0.1
Prothrombin	1.21	0.009	0.1
Serotransferrin	1.24	0.03	0.2
Serpin family G member 1	0.89	0.04	0.2
Vitronectin	0.75	0.02	0.1

^a,b^Different isomers of the same protein, ^c^bactericidal/permeability-increasing protein, ^d^Immunoglobulin, ^e^alpha-1-microglobulin/bikunin precursor.

**Table 6:** Significant changes in plasma protein levels at 2.5 years of age, compared with 1.5 years of age, in 16 Standardbred horses in training. Fold change values > 1 indicate that 2.5-year-olds had higher levels than 1.5-year-olds, values < 1 indicate that 2.5-year-olds had lower levels than 1.5-year-olds.

Protein	Fold change	*p*-value	FDR-adjusted *p*-value
Angiotensinogen	1.21	0.01	0.4
Apolipoprotein A-II	1.48	0.0002	0.02
Apolipoprotein H	1.17	0.03	0.4
BPI^a^ fold containing family A member 2	0.38	0.01	0.4
Carboxypeptidase B2	1.28	0.04	0.4
Carboxypeptidase N subunit 2	1.33	0.05	0.5
Ceruloplasmin	0.87	0.02	0.4
Clusterin	1.30	0.04	0.4
Fetuin B	2.02	0.00009	0.01
Glutathione peroxidase	0.84	0.03	0.4
Interleukin 1 receptor accessory protein	1.58	0.002	0.08
Kininogen 1	1.30	0.02	0.4
Prothrombin	1.22	0.03	0.4
Serpin family G member 1	0.84	0.03	0.4

^a^Bactericidal/permeability-increasing protein.

**Table 7:** Significant changes in plasma protein levels at 3.5 years of age, compared with 1.5 years of age, in 16 Standardbred horses in training. Fold change values > 1 indicate that 3-year-olds had higher levels than 1.5-year-olds, values < 1 indicate that 3-year-olds had lower levels than 1.5-year-olds.

Protein	Fold change	*p*-value	FDR-adjusted *p*-value
Adipsin	1.08	0.05	0.2
Alpha-1-antitrypsin	0.64	0.01	0.08
Alpha-2-glycoprotein 1, zinc-binding	1.32	0.003	0.04
Alpha-2-macroglobulin	0.82	0.02	0.09
Angiotensinogen	1.18	0.009	0.07
Antithrombin-III	1.14	0.03	0.1
Apolipoprotein A-II	1.71	0.00003	0.002
Apolipoprotein H	1.26	0.0006	0.01
BPI^a^ fold containing family A member 2	0.32	0.003	0.04
Carboxylic ester hydrolase	0.73	0.05	0.2
Carboxypeptidase N subunit 2	1.37	0.007	0.07
CD5 molecule like	1.43	0.00006	0.003
Ceruloplasmin	0.84	0.002	0.03
Clusterin	1.56	0.0002	0.005
Coagulation factor V	1.23	0.05	0.2
Complement C1q C chain	0.79	0.01	0.08
Complement C4-A	0.84	0.02	0.1
Complement C7	1.28	0.02	0.1
Complement factor I	1.27	0.03	0.1
Complement subcomponent C1r	0.83	0.01	0.08
C-type lectin domain family 3 member B	1.62	0.04	0.2
Fetuin B	2.38	5.0E-10	7.1E-08
Gc^b^-globulin	1.10	0.05	0.2
Haemoglobin subunit alpha	2.97	0.04	0.2
Haemoglobin subunit beta	2.86	0.02	0.1
Histidine-rich glycoprotein	1.25	0.03	0.1
Hyaluronan binding protein 2	1.37	0.04	0.2
Ig^c^ gamma 1 heavy chain constant region	0.77	0.03	0.1
Ig^c^ heavy constant mu	1.17	0.007	0.07
IGF binding protein acid labile subunit	0.71	0.01	0.08
Ig^c^-like domain-containing protein	1.45	0.02	0.1
Ig^c^-like domain-containing protein	1.71	0.05	0.2
Interleukin 1 receptor accessory protein	1.66	0.008	0.07
Joining chain of multimeric IgA and IgM	1.43	0.005	0.05
Kininogen 1	1.56	0.0002	0.006
Leucine-rich alpha-2-glycoprotein 1	1.38	0.01	0.08
Lumican	0.54	0.002	0.02
Peptidoglycan recognition protein 2	0.73	0.04	0.2
Plasminogen	1.21	0.02	0.09
Protein AMBP^d^	1.35	0.03	0.1
Prothrombin	1.37	0.001	0.02
Serpin family A member 6	0.72	0.03	0.1
Serpin family G member 1	0.71	0.0002	0.007

^a^Bactericidal/permeability-increasing protein, ^b^group-specific component, ^c^Immunoglobulin, ^d^alpha-1-microglobulin/bikunin precursor.

## Discussion

To our knowledge, this is the first study to apply proteomics to plasma samples from young horses fed and trained under standardised conditions for two years, until fit for racing at the age of 3.5 years. The horses were subjected to two different training programmes that have been shown to cause significant differences in circulatory responses by the age of 2 years, i.e. lower heart rate during and after exercise and elevated exercise haematocrit in the High group compared with the Low group^12^. These alterations are expected training responses in horses^14^. Interestingly, proteomics analysis did not reveal any differences between the training groups when FDR adjustment was applied. The strategy with strict statistical evaluation and multiple testing correction is always recommended to avoid false positive findings. However, strict statistical analysis could be argued to be considered too conservative for this type of study, given that several of the proteins found in plasma are interrelated and parts of the same biochemical process^15^. One could from this perspective use a gene set enrichment analysis (GSEA) methodology, also called functional enrichment analysis or pathway enrichment analysis. We applied a hypothesis-generating approach, and therefore we have selected to also comment on the 16 proteins that differed between the age groups at some time point based on the t-test only. The proteins found in higher levels in High group plasma compared with Low group plasma were an unknown protein and serotransferrin. Serotransferrin (also called transferrin) is an iron-binding transporter protein mainly produced in the liver^16^. The elevated level could be associated with the higher haematocrit observed in the High group^12^. It is known since long that training increase the blood haemoglobin content and the red cell reservoir of the spleen in horses^17,18^. In a study on humans, higher plasma serotransferrin levels at rest have also been observed in trained subjects compared with untrained^19^. However, this contradicts findings in a previous study in Thoroughbreds, where transferrin levels decreased during a 90-day training programme^20^. The pre study fitness level of these horses was however not clear and no changes were observed in haemoglobin concentrations either.

Proteins present in lower levels in plasma in the High group compared with the Low group at some time point were afamin, apolipoprotein (Apo) E and H, extracellular matrix protein 1 (ECM1), fibulin-1, Ig heavy constant mu, Ig lambda light chain variable region, protein alpha-1-microglobulin/bikunin precursor (AMBP), serpin family D and F member 1, complement factor H (CFH), complement factor properdin (CFP) and haptoglobin. The exact functions of these proteins are unknown, but several are associated with fat metabolism and oxidant/antioxidant processes, the cardiovascular system, bone formation and inflammation. Apo E binds to the surface of chylomicron, very-low-density lipoprotein and high-density lipoprotein (HDL), where it has a role as a regulator in their metabolism^21^. The function of Apo H (also called beta 2-glycoprotein 1) is still unclear, but it has been implicated in lipid metabolism and blood coagulation^22^. It has been suggested that high intensity exercise is a trigger for clot formation in humans, although the mechanism is unknown^23^, and perhaps more adaptations to avoid hypercoagulability was occurring in the High group. Afamin is a glycoprotein with similar structure to albumin, but knowledge of its function is limited^24^. It has been shown in human plasma studies that afamin binds to vitamin E^25^and is involved in transportation of vitamin E across the blood brain barrier^26^. It is possibly also involved in osteoblast metabolism and bone formation^27,28^. Haptoglobin is involved in protection against haem-mediated inflammation and oxidative damage^29^. The observed reduction in plasma haptoglobin levels contradicts findings in a study monitoring 90 Thoroughbreds for around one year, where no differences in haptoglobin levels were seen^30^. It also contradicts findings in a study of 17 Thoroughbreds subjected to a standardised training programme for six months, where an increase was observed^31^. Protein AMBP is primarily synthesised in the liver and is the precursor for alpha-1-microglobulin (A1M) and bikunin, proteins that have different structures and functions in the body^32^. A1M is an antioxidant with reductase activity and haem- and radical-binding functions in most intracellular, intravascular and extravascular regions in the body^32^. Bikunin is a protease inhibitor which can be found in blood and tissue alone or in complex with heavy chain proteins^33^, and it inhibits complement system activation, has extracellular matrix protective activity and is involved in cell regulation^33^. CFH and CFP are both regulators in the alternative pathway of the complement system, but CFH is a negative and CFP a positive regulator^34–36^. ECM1 is a glycoprotein with a double helix structure similar to albumin^37^and is involved in angiogenesis^38^, skin differentiation, integrity and homeostasis^37,39^, and possibly also in endochondral bone formation^40^. Fibulin-1 is an extracellular matrix protein associated with fibronectin and is found in most organs^41^. It has a form circulating in the blood, but its specific function and site of synthesis are still unknown^41^. Serpin family D member 1 (also called heparin cofactor II) is mainly synthesised in the liver. Its physiological role is not fully understood, but it is known to inhibit thrombin, the predominant coagulation protease^42^. Serpin family F member 1 (also called pigment epithelium-derived factor, PEDF) is synthesised throughout the body with diverse functions (e.g. anti-inflammatory, antioxidant, anti-angiogenetic) and is involved in bone homeostasis^43,44^. A decrease in Ig heavy constant mu and Ig lambda light chain variable region indicate alterations in the immune system.

Although the exact functions of the proteins discussed above are in some cases unknown, our findings indicate that an increased high intensity training distance will improve iron transporting capacity and alter the cardiovascular response, which is in accordance with physiological observations reported earlier in these horses^12^. Furthermore, a longer exercise distance appears to alter fat metabolism and oxidant/antioxidant processes, bone formation and inflammation processes. Increased activity of fat metabolism (3-hydroxyacyl-CoA-dehydrogenase) with training has been observed earlier in Standardbred horses^45^. If energy metabolism is shifted towards aerobic metabolism, the need for regulation of oxidative and antioxidative processes is also altered. Changes in reactive oxygen species and “oxidative stress” have been observed following acute intensive exercise in horses^46^ and our results are therefore not unexpected.

### Changes over time

When changes over time were analysed (irrespective of training group), the level of 90 proteins differed at some time point according to the t-test, but after FDR adjustment only 17 protein levels remained significantly different at any time point compared with at 1.5 years of age. The proteins that showed higher plasma levels (after FDR adjustment) at some time point compared with age 1.5 years were Apo A-II and H, ECM1, fetuin B, alpha-2-glycoprotein 1 zinc-binding (AZGP1), CD5 molecule like, clusterin, joining chain of multimeric IgA and IgM, kininogen 1 and prothrombin. Alterations in several of these proteins indicate that similar systems, processes and tissues were affected over time as with training, i.e. fat metabolism and oxidant/antioxidant processes, bone formation, circulation and the immune system. Apo A-II is a key regulator of HDL metabolism and structure^47^and, as mentioned, ECM1 is involved in angiogenesis^38^. Both fetuin B and kininogen 1 belong to the cystatin family and are synthesised in the liver^48^. Fetuin A is involved in systemic inflammation, regulation of mineralisation and insulin, while Fetuin B has a similar tissue distribution, and is suggested to have similar functions, as Fetuin A^49^. Kininogen has several splice variants that are involved in blood clotting and angiogenesis and is also a precursor for the kinin hormones^48,50^. Kinins are short-lived mediators with a range of functions in the body, such as induction of vasodilation and smooth muscle contraction, enhanced capillary permeability, potent inflammatory mediator, stimulating production of superoxide radicals and nitric oxide, regulating renal function and blood pressure, and involvement in cardiac homeostasis^51^. Clusterin (also called Apo J) is a multifunctional glycoprotein which is present in almost all fluids in the body and in some locations also in the intracellular matrix^52^. It has very varied functions, such as involvement in inflammation, cardio- and neuroprotection, satiety and hunger, and apoptosis^52^. Prothrombin is synthesised in the liver and plays a major role in coagulation and haemostasis, but is also involved in regulation of endothelial cell proliferation, regulation of inflammation, wound healing and as a precursor for thrombin, which is involved in coagulation^53^. AZGP1 has a similar structure to MHC 1 and is involved in lipid and glucose metabolism and insulin sensitivity^54^. Increases in CD5 molecule like and joining chain of multimeric IgA and IgM indicate alterations in the immune system.

Proteins that showed lower plasma levels at some time point compared with age 1.5 years were haemopexin, BPI fold containing family A member 2 (BPIFA2), ceruloplasmin, lumican and serpin family G member 1. Haemopexin is similar in function to haptoglobin, i.e. it is involved in the binding and clearance of haem^29^. Serpine family G member 1 (also called C1 inhibitor) is a protease inhibitor involved in regulation of vascular permeability and in anti-inflammatory functions^55^. Ceruloplasmin is mainly synthesised in the liver and can catalyse oxidation of a number of different molecules^56^. The best-known of these is oxidation of ferrous iron, which makes it possible for iron to bind to the transporter protein transferrin^57^. Lumican is a small leucine-rich proteoglycan which is involved in the maintenance of tissue homeostasis and cell proliferation, migration and differentiation^58^. More specifically, it is involved in wound healing^59^, in the regulation of collagen fibrillogenesis^58^, has anti-angiogenesis function^60^and is possibly also involved in muscle^61^and bone formation^62^. BPIFA2 (also called parotid secretory protein, PSP) is a salivary protein that can bind to bacterial lipopolysaccharide^63^and it has been shown to have antibacterial functions^64^, but many of its functions are still unknown.

It is evident that many of the proteins mentioned above have functions within the immune system. In humans it well accepted that prolonged, intense exercise causes an “open window” of immunodepression during the recovery phase^65^. Also in horses, acute exercise may induce phenomena linked to immunosuppression for hours and days post exercise^66–68^but studies on long term effects of systematic training on immune function are scares. However, it has been suggested that high intensity training can be associated with e.g. a reduction in neutrophil function^69^. In a study where plasma samples collected at rest from two groups of Thoroughbred horses (athletic vs. sedentary) were compared it was also shown that genes involved in inflammatory process, such as TLR4, IL1b, IL1RII, IL18 and IL6 were expressed to a greater extent in the athletic group^70^. The authors suggested that a focused training regime increase the baseline expression of genes involved in the inflammatory process which could support a prompt response to exercise induced stress. More studies are needed to better understand the effect of systematic high intensity training on immune function and the susceptibility for e.g. respiratory disease.

The above results also indicate that changes in plasma proteins related to energy metabolism, bone formation and circulatory functions occurred in the young Standardbred horses in training during the study. This was not unexpected based on earlier observations on these horses of body development (skeletal growth), metabolic changes (improved lactate threshold) and physiological responses (lowered heart rate, increased haematocrit, lower diastolic blood pressure)^11,12^. It is also known that bone morphology changes and bone density increases with training in Standardbred horses^71,72^. The finding that bone growth was stimulated by the training programmes is supported by the interrupted time-dependent decline in insulin-like growth factor 1 (which targets bone and cartilage) observed previously in these horses after high-intensity training was introduced^73^.

A previous study of horses during a single session of endurance exercise concluded that such exercise affects plasma proteins involved in pathways related to oxidant/antioxidant activity, inflammation, coagulation, immune modulation and cellular and vascular damage^5^. We obtained similar findings, although we studied effects of long-term training. As in the previous study^5^and discussed above, we found that several alterations in the plasma protein profile were related to the immune system, inflammation, clotting and wound healing. However, in our horses these observations might also have been linked to factors other than exercise, e.g. some horses lost days to training and in some cases this was due to orthopaedic problems (mainly joint inflammation)^74^. In addition, almost all horses had wounds and mud fever at least once during the study (wounds mostly observed after physical interactions between individuals in the group housing system)^74^. By the end of the study (age > 3 years), some horses had health problems such as mud fever, wounds, fractures (trauma) and joint inflammation, which could be expected to be reflected in the plasma proteomic profile. Moreover, from September to November some horses were observed coughing and a decrease of *≥* 0.5 °C in weekly mean body temperature was observed in nine horses (data not shown). Lowering of body temperature is a phenomenon commonly discussed among horse trainers and is associated with impaired performance. It is possibly also reflected in the level of plasma proteins related to immune functions.

Nine horses were fit to race during the study and at least 12 continued a racing career with other trainers when the study was completed^75^. One horse won two races already during the study and later became by far the most successful of all experimental horses (101 races and earnings > 1 million SEK by 18 November 2023). The last blood sample in this study (December, age 3.5 years) was collected 38 days after victory number one and 15 days before victory number two, so from a performance perspective it is of interest to analyse the proteomic profile of this exceptional horse. On ranking all horses and protein analyses in the December sample from highest to lowest, this horse was distinguished by having the highest plasma level of clusterin, the next highest level of apolipoprotein A-II and the third highest plasma level of protein AMBP, combined with low levels of CFP, extracellular matrix protein and fibulin-1. The three compounds found in elevated concentrations increased over time in all horses (see above) and may therefore be candidate biomarkers for good performance. CFP concentration was not only low in this horse, but also lower in all horses compared with age 1.5 years. This protein regulates a complement pathway of the innate immune system and accordingly is part of the first line of defence against microorganisms^36^. This observation may therefore simply show that a microbial infection was not ongoing in this particular horse. Concerning extracellular matrix protein, a comparatively low level was also observed in High group horses at 2 years of age, when cardiovascular fitness was higher in this group^12^, indicating that it might be associated with fitness. As mentioned, the specific function of fibulin-1 is unknown (association with extracellular matrix structures) and the importance of the low level in blood plasma remains to be determined.

The plasma proteomic analysis was unable to identify significant differences between horses in the two high-intensity training programmes if p-values were FDR-adjusted, but revealed many significant changes in the horses over time. The lack of differences between the training groups in terms of plasma proteomic response is surprising, since physiological differences have been documented for the horses^12^. However, it might be because compliance with the training schedule deteriorated after the first 0.5 year of high-intensity training (the High group started to have relatively more days lost to training), so that by the age of 3.5 years, total lifetime training distance did not differ between the High and Low groups^74^. Loss of training days by the horses was mainly based on the trainer’s decision, i.e. horses which were not considered fit to train were omitted from a training session. Methodological limitations may also partly explain why no differences were observed, e.g. LC-MS techniques suffer from ion suppression, with negative effects on detection capability, accuracy and precision^76^, creating problems in analysis of low-abundance proteins.

Another potential limitation is that the samples analysed had been frozen for 9–11 years, which may have affected the results. However, in order to investigate if freeze drying of the samples had occurred the total plasma protein concentration was analysed and values within normal ranges were observed (65–75 g/L). The same samples have also been used in studies of IGF-1^73^and metabolomics^77^and levels observed were within the range reported previously^78^, which implies that samples were well preserved during storage.

Another limitation of this study is the lack of a group of horses of the same age subjected to no exercise at all. Although the main aim of the study was to explore differences in the proteomic profile in response to two different training programmes, where the High group could be considered the control treatment (comparable to standard practise^11^), a third non-exercised group would have added interesting information on the effects of growth and aging only. A non-exercised group was also part of the initial experimental plans but it was not possible to realize.

In conclusion, proteins involved in pathways related to energy metabolism, red cell metabolism, circulation, oxidant/antioxidant activity, bone formation, inflammation, immune modulation and cellular and vascular damage were found to be affected in growing Standardbred trotters in training. Our study also indicate that proteomics analysis of blood plasma could be a viable tool for evaluation of exercise adaptations, performance and for health monitoring, with several potential biomarkers identified. Further studies are however, needed to validate our findings and to uncover the mechanisms involved. Nevertheless, we suggest that future studies on biomarkers for improved performance could start with focus on clusterin, apolipoprotein A-II and protein AMBP.

## Electronic supplementary material

Below is the link to the electronic supplementary material.

Supplementary Material 1

Supplementary Material 2

## Data Availability

The dataset used and analysed in this study is found in Supplement 2.
